# Assessment of Tooth Preparations Submitted to Dental Laboratories for Fabrication of Monolithic Zirconia Crowns

**DOI:** 10.3390/dj9100112

**Published:** 2021-09-27

**Authors:** Ramtin Sadid-Zadeh, Hadjer Sahraoui, Brian Lawson, Robert Cox

**Affiliations:** 1 Department of Restorative Dentistry, University at Buffalo School of Dental Medicine, Buffalo, NY 14214, USA; 2 Department of Prosthodontics, University at Texas Health Science Center, San Antonio, TX 77030, USA; hadjersa@buffalo.edu; 3 Department of General Dentistry, New York University Langone Dental Medicine, Nashville, TN 37011, USA; brianlaw@buffalo.edu; 4 Department of Pediatric Dentistry, University at Buffalo School of Dental Medicine, Buffalo, NY 14214, USA; rgcox@buffalo.edu

**Keywords:** CAD-CAM, quality of tooth preparation, monolithic zirconia

## Abstract

Purpose: The objective of this study was to assess the quality of posterior teeth prepared for monolithic zirconia crowns. Materials and Methods: A total of 392 STL-files of posterior preparations for monolithic zirconia crowns were evaluated in this study. Three-dimensional (3D) images were evaluated using a software (3D Viewer; 3Shape A/S, Copenhagen, Denmark) for finish line design, finish line width, occluso-cervical dimension, total occlusal convergence (TOC), intercuspal angulation, finish line quality, line angle form, and presence or absence of undercut at the axial wall and unsupported lip of enamel. The assessment was performed by two calibrated evaluators. Then, data were descriptively analyzed. Data for occluso-cervical dimension and TOC were descriptively analyzed according to their location. Results: Thirty-nine percent of premolars, 77% of first molars, and 91% of second molars had an average occluso-cervical dimension of less than 3 mm (premolars) and 4 mm (molars), with most of the preparations having a TOC of more than 20 degrees. More than 50% of preparations had undercut, unsupported enamel and/or unacceptable finish line quality. Conclusions: The quality of tooth preparation including finish line quality, absence of unsupported enamel and undercut at the axial wall should be evaluated when preparing monolithic zirconia crowns.

## 1. Introduction

Historically, dental practitioners have employed a wide variety of materials when fabricating complete coverage crowns. In the last decade, however, ceramic restorations have increasingly become the material of choice for use in fixed prosthodontics [1]. For example, while 65% of the single-unit fixed restorations were fabricated from metal-ceramic in 2007, only 20% of the restorations manufactured by one of the largest dental laboratories in the United States were metal-ceramic in 2012 [2]. Moreover, because of their high strength, wear compatibility with natural dentition, esthetic appeal, and low cost, multiple sources report a large increase in the use of all-ceramic restorations during the past decade [3,4]. Currently, 32% of dental practitioners select monolithic zirconia as the material of choice for use in complete coverage restoration of posterior teeth [5]. Moreover, at one of the largest laboratories in the United States, 75% of complete coverage crowns are fabricated from monolithic zirconia [6].

Similar to conventional complete coverage crowns, the longevity of monolithic zirconia crowns is affected by the principles of tooth preparation such as total occlusal convergence (TOC), the occluso-cervical/inciso-cervical dimensions, the presence or absence of undercut, the amount of occlusal reduction, finish line form and depth, line angle form, and surface texture [7]. However, for computer-aided design/computer-assisted manufacture (CAD-CAM) fabricated crowns, such as those fabricated from monolithic zirconia, additional principles, including the quality of the finish line form, line angle form, and the occlusal surface preparation design (anatomical, semi-anatomical, or non-anatomical), become essential [8,9].

The TOC contributes to the retention of a preparation. While numerous sources recommend a TOC ranging between two to six degrees for optimal retention of a preparation, laboratory evaluations suggest the TOC of preparations should be between six and twelve degrees [10,11,12]. However, a TOC range between 10 and 20 degrees with no undercut on the axial wall along with a minimum occluso-cervical dimension of 4 mm for molars and 3 mm for premolars and incisors is recommended [7]. Recent studies show that the guideline for TOC is followed by dental students while at the school, but the guideline is missed by dental practitioners [13,14]. Moreover, studies have shown that an increased TOC from 12 and 20 degrees may impact the fracture resistance and internal adaptation of CAD-CAM crowns [15,16].

The resistance to fracture of all-ceramic crowns is dependent on the ceramic material used, crown thickness, the tooth position in which the restoration will be luted, finish line design, and, in cases employing bilayer ceramics, core/veneer bond strength [17,18,19]. The suggested amount of reduction when using monolithic lithium disilicate crowns ranges from 1.5 mm to 2 mm [20]. However, monolithic zirconia has a higher flexural strength than lithium disilicate, allowing for minimal tooth preparation [21]. Given that the flexural strength of monolithic zirconia ranges between 900 to 1600 MPa, depending on the mechanical properties of zirconia used, manufacturers recommend tooth reduction of 0.6 mm or more for posterior monolithic zirconia crowns [22,23]. A recent study showed that monolithic zirconia crowns with 0.6 mm thickness had a fracture load comparable to monolithic lithium disilicate restorations with 1.5 mm and 2 mm thickness [24]. Moreover, a study from Nakamura et al. showed that monolithic zirconia crowns with a 0.5 mm occlusal thickness have a higher fracture load than a monolithic lithium disilicate crown with a 1.5 mm occlusal thickness [25]. An additional study confirmed that monolithic zirconia crowns with a 0.5 mm thickness withstand occlusal forces [26].

Finish line design and depth may impact the marginal adaptation and fracture load of monolithic zirconia. The data from different studies are conflicting for the selection of a chamfer or modified shoulder; nevertheless, the results from these studies present a clinically acceptable marginal adaptation regardless of finish line design [27,28,29]. A comparative study showed there is no significant difference in the fracture load of zirconia crowns fabricated on a 0.5, 0.7 or 1.0 mm chamfer depth [25]. However, in a 10-year simulated mouth environment, Mitov et al. showed that a conservative finish line width may have a positive influence on the fracture load of zirconia crowns [30]. This study suggested that feather edge and 0.4 mm chamfer finish line designs have a significantly higher fracture load compared to 0.8 mm chamfer finish line designs [30]. Considering that a thin margin provides enough fracture load resistance for monolithic zirconia, the decision of finish line depths should be based on the minimal tooth structure removal required to develop a physiologic emergence profile and to provide esthetics depending on the natural die material shade. In addition, a rough, irregular, or stepped finish line increases the margin length and, subsequently, reduces marginal adaptation of restorations [11]. Importantly, because cutting tools in milling machines may not mill a designed rough margin due to limitations of the cutting tool diameter, a smooth finish line becomes vital for CAD-CAM crowns [31].

The purpose of creating a round line angle form with conventionally fabricated crowns is to facilitate laboratory procedures and to optimize the internal fit of restorations [7]; additionally, preparations with a round line angle form minimize the stress concentration on all-ceramic crowns [32,33,34]. Moreover, well-rounded line angle forms are required for CAD-CAM fabricated restorations because of the cutting tool diameter used to mill the intaglio surface of the restorations. When utilizing a milling machine that uses a 1.0 mm diameter cutting tool for removing the intaglio surface of the restoration, a sharp line angle form leads to an ill-adapted restoration both at the margin and internally [35].

The studies regarding the impact of the tooth preparation surface texture on the marginal fit and retention of conventionally fabricated crowns are inconsistent; nevertheless, there is general agreement that some level of surface coarseness improves the retention of crowns [26,33,34,35,36,37,38]. In contrast, a study by Sinhori et al. demonstrated that the surface texture of tooth preparation does not have any impact on the marginal and internal fit of CAD-CAM fabricated crowns [39]. However, more studies are needed to evaluate the impact of preparation surface texture on the marginal and internal fit, and retention of different CAD-CAM crowns.

When preparing a tooth for CAD-CAM crown, modifications to the classic tooth preparation design are needed to compensate for the milling process. A simple practical occlusal reduction design with a non-anatomical occlusal surface has been suggested for CAD-CAM restorations. Such a design may reduce milling times, improve accuracy, and expedite the treatment process, all of which will enhance the overall quality of CAD-CAM crowns [9,40]. However, two recent studies have demonstrated that preparing a non-anatomical occlusal surface leads to a significantly larger marginal gap on CAD-CAM crowns [9,41]. Preparing non-anatomical occlusal surface results in more tooth structure loss, a factor that may influence the resistance and retention form of the preparation [41].

Errors in a tooth preparation are reported as a laboratory challenge second to having an inaccurate impression [42]. Evaluating how far tooth preparations performed by general practitioners stray from the ideal has been the subject of several investigations [43,44]. Multiple studies have evaluated tooth prepared for lithium disilicate and zirconia-based CAD-CAM crowns; however, there is no information regarding the adherence to these principles when practitioners prepare a tooth for a monolithic zirconia crown [14,45]. Considering an increased use of monolithic zirconia crowns for patient treatment, the purpose of this study was to evaluate digital models from tooth preparations that had been submitted to local laboratories for use in the fabrication of posterior tooth monolithic zirconia crowns.

## 2. Materials and Methods

This study was conducted in accordance with the Declaration of Helsinki (1964), as revised in Venice in 1983, and received an exemption (STUDY00002820) from the Institutional Review Board of the University at Buffalo School of Dental Medicine. Nineteen dental laboratories in Buffalo, NY, USA were identified using an online search engine (Google) and were contacted to determine whether they prominently use CAD-CAM technology for crown fabrication. Three out of 19 dental laboratories participated in this study.

Inclusion criteria were posterior tooth preparations submitted for monolithic zirconia, whether they were scanned using an intraoral or desktop scanner. Exclusion criteria were if preparations of anterior teeth, preparation of posterior teeth for layered zirconia, or other materials except for zirconia. A total of 392 standard tessellation language (STL) files from prepared teeth that had been submitted for fabrication of monolithic zirconia crowns between 1 May and 1 June 2017 were received from the participating laboratories and evaluated in this study. The preparations were assessed by two calibrated evaluators using 3D viewer software (3Shape A/S; Copenhagen, Denmark) for finish line design, finish line width, occluso-cervical dimension, total occlusal convergence (TOC), intercuspal angulation, finish line quality (rough, irregular, or stepped finish line), line angle form, and the presence or absence of undercut and unsupported lip of enamel.

Finish line design of each preparation was identified by visual assessment of 3-dimensional (3D) images of preparations and reported as chamfer, shoulder, modified shoulder, or feather edge. Finish line width was measured at eight locations: mid-mesial, mid-distal, mid-buccal, mid-lingual, and mesio-buccal, disto-buccal, mesio-lingual, and disto-lingual line angles. Then, the values were averaged to represent the width of the entire preparation. The finish line width was evaluated using the digital ruler incorporated into the 3D viewer software (3Shape A/S); the values were reported in millimeters. The presence/absence of a rough, irregular, or stepped finish line (the finish line quality) and the presence/absence of unsupported lip of enamel were also assessed visually for the entire preparation on 3D images.

In order to evaluate TOC and occluso-cervical dimension of preparations, STL files were sectioned at mid-buccolingual and mid-mesiodistal in the 3D viewer software (3Shape A/S) and the angulation between the buccolingual and mesiodistal axial walls was measured using the Loma Linda TOC guide. Buccolingual and mesiodistal TOCs were then reported as greater than 20 degrees or equal to/less than 20 degrees. The occluso-cervical dimension was measured using the digital ruler that was incorporated into the software. The occluso-cervical dimension was reported as ideal when the average was greater than or equal to 3 mm for premolars and 4 mm for molars. The occlusal intercuspal angulation was measured buccolingually using a protractor, and measurements were reported as non-anatomical when the intercuspal angulation was approximately 180 degrees; otherwise, the angulations were considered anatomical. Moreover, 3D images of the preparations were examined for the presence/absence of undercut at the axial wall and acceptable/unacceptable line angle form (cusp sharpness). Data gathered from this study were reported descriptively.

## 3. Results

**Figure 1:**
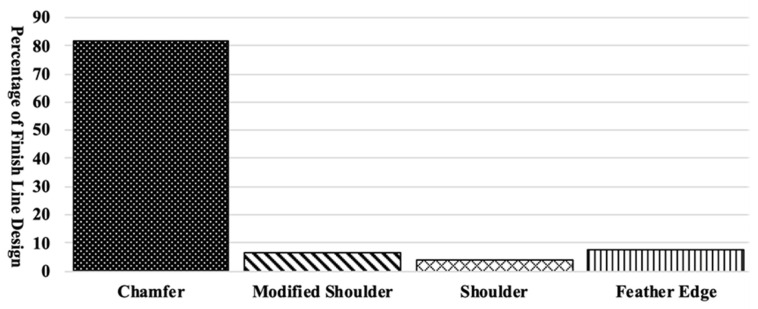
Percentage of each finish line design on teeth prepared for monolithic zirconia crown.

A total of 392 posterior tooth preparations were evaluated in this study. Posterior tooth preparations consisted of 113 premolars, 172 first molars, and 107 second molars. The finish line width ranged between 0.0 and 2.3 mm with a mean of 0.62 mm (±0.2); a comparison of different finish line designs showed that chamfer design accounted for 82% of the finish line designs (Figure 1).

**Figure 2:**
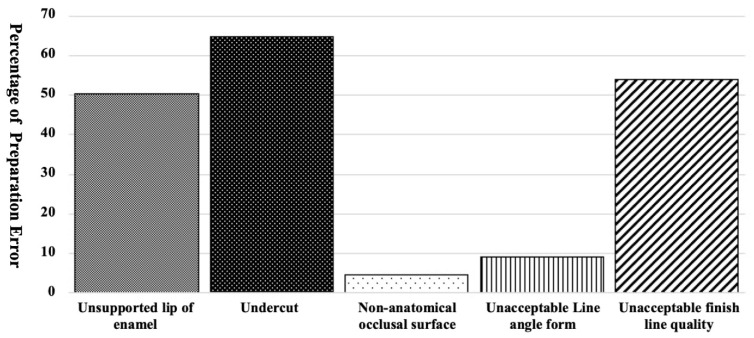
Percentage of preparations with unsupported lip of enamel, undercut at the axial wall, a non-anatomical occlusal surface, and an unacceptable line angle form or finish line quality.

Figure 2 shows the percentage of preparations with an unsupported lip of enamel, undercut at the axial wall, a non-anatomical occlusal surface, and an unacceptable line angle form or finish line quality. Most preparations presented with an unsupported lip of enamel, undercut at the axial wall, or an unacceptable finish line quality. The above-mentioned errors were accounted for 50%, 65%, and 54% of preparations, respectively.

Finally, the distribution of posterior teeth prepared with unacceptable occluso-cervical dimensions and axial wall height based on their intraoral region were analyzed (Table 1). A total of 113 premolars, 172 first molars, and 107 second molars were evaluated. Thirty-nine percent of premolars had an average occluso-cervical dimension of less than 3 mm. However, an occluso-cervical dimension of less than 4 mm was observed in 77% of first molars and 91% of second molars. Total occlusal convergence of more than 20 degrees was present in 80% of premolars, 95% of first molars, and 91% of second molars.

## 4. Discussion

Tooth preparation is a major factor influencing the longevity of an indirect restoration. In addition to principles of tooth preparation required for conventional restorations, factors such as a rough, irregular, or stepped finish line and a non-anatomical occlusal surface that may lead to an increased marginal gap must be considered when preparing teeth for CAD-CAM crowns [9,11,31,41]. An unsupported enamel lip, which was observed in 50% of the preparations, may lead to fracture of the enamel prism at the margin during or after cementation; such a fracture can lead to marginal opening and potential secondary caries [10]. Additionally, a rough, irregular, or stepped finish line is detrimental for the fabrication of CAD-CAM crowns; however, preparations with poor finish line quality were observed in 46% of the preparations analyzed in this study. Cutting tools in CAD-CAM milling machines have limitations in their diameter, as a result, a smooth finish line quality is essential to fabricate a well-adapted CAD-CAM crown [31].

Finish line width and design are determined by the minimal thickness of the restorative material that provides sufficient strength and by the minimal space required to develop a physiologic emergence. In addition, finish line design may have an impact on the marginal gap and fracture strength of the CAD-CAM restorations. Studies conducted in vitro report that finish line design does not influence the marginal gap for monolithic zirconia crowns [27,28,29]. However, a conservative finish line width increases the fracture strength of monolithic zirconia [30]. Mean finish line width reported in this study was 0.62 mm, with the finish line width ranging from 0.0 mm to 2.3 mm. Because a wide finish line width does not provide higher fracture strength to monolithic zirconia crowns, practitioners should preserve the tooth structure by preparing a conservative finish line width unless the principles of tooth preparations for esthetics are compromised.

A TOC range between 10 and 20 degrees, with a minimum occluso-cervical dimension of 4 mm for molars and 3 mm for premolars, is recommended for long-term retention and resistance of restorations [7]. In this study, 70% of teeth preparations had the minimum recommended occluso-cervical dimension, and 89% of preparations had a TOC of more than 20 degrees. Data for TOC in this study was in agreement with Winkelmeyer et al. study showing preparations performed by dental practitioners exceeded the recommended TOC; however, this data differed from the Tiu. et al. study results [13,41,45]. This difference in data from Tiu et al. study might be because they evaluated preparations performed by dental students while the principle of tooth preparation is reinforced during their education [13]. The occluso-cervical dimension was not met the suggested dimension in 30% of preparations. The combination of a short occluso-cervical dimension and large TOC angulation should be avoided as it impacts the resistance form of the preparation. While the choice of adhesive resin luting agent, along with sandblasted zirconia and application of the appropriate primer, increases the retention of zirconia crowns, this bonding may degrade with aging [9,46]. As a result, practitioners should follow the recommended occluso-cervical dimension and TOC to provide the appropriate retention and resistance form for the restorations.

In this study, 67% of the preparation lacked a correct path of placement due to the undercut at the axial wall. Available design software is equipped with a tool blocking the undercut detected on the 3D image of tooth preparation; however, if the digital design technicians misuse the blocking tool due to the presence of the undercut, it may result in a larger cement space. In addition, preparations with an inappropriate line angle form may lead to excessive removal of the internal surface of the crown, causing a large cement space; this outcome was observed in 9% of the preparations. Overall, the increase in cement thickness may lead to restoration with significantly lower fracture strength [47,48].

The limitations of this study are that only CAD-CAM crown preparations using monolithic zirconia were evaluated and that the data were gathered from a limited number of laboratories. In addition, the finish line width was only measured at 8 locations. Future studies should use software with the capability of measuring the entire finish line width. Moreover, future studies should be examining data from a broader geographical area, and different materials used for CAD-CAM crown preparation are required.

## 5. Conclusions

Within the limitations of this study, continuing education for dentistry practitioners should emphasize adherence to the principles of tooth preparation when using CAD-CAM crowns in order to improve the quality of preparation and long-term survival of the restorations. The principles such as a smooth finish line, absence of unsupported enamel, and undercut at the axial wall should be emphasized in education.

## Figures and Tables

**Table 1:** Number of prepared teeth evaluated for each tooth and number of unacceptable OCD and TOC for each location.

	Max. Premolar	Mand. Premolar	Max. 1st Molar	Mand. 1st Molar	Max. 2nd Molar	Mand. 2nd Molar	Total
Number of evaluated preparations	79	34	72	100	45	62	392
Number of preparations with unacceptable OCD	27	17	63	69	37	60	273
Number of preparations with unacceptable TOC	59	31	67	96	42	55	350

Max. = Maxillary, Mand. = Mandibular, OCD = Occluso-cervical Dimension, TOC = Total Occlusal Convergence.

## Data Availability

Not applicable.
